# Carbapenems to Treat Multidrug and Extensively Drug-Resistant Tuberculosis: A Systematic Review

**DOI:** 10.3390/ijms17030373

**Published:** 2016-03-12

**Authors:** Giovanni Sotgiu, Lia D’Ambrosio, Rosella Centis, Simon Tiberi, Susanna Esposito, Simone Dore, Antonio Spanevello, Giovanni Battista Migliori

**Affiliations:** 1 Clinical Epidemiology and Medical Statistics Unit, Department of Biomedical Sciences, University of Sassari—Research, Medical Education and Professional Development Unit, AOU Sassari, Sassari 07100, Italy; gsotgiu@uniss.it (G.S.); simonedore@hotmail.com (S.D.); 2 World Health Organization Collaborating Centre for Tuberculosis and Lung Diseases, Fondazione S. Maugeri, IRCCS (Istituto di Ricovero e Cura a Carattere Sceintifico), Via Roncaccio 16, Tradate 21049, Italy; lia.dambrosio@fsm.it (L.D.A.); rosella.centis@fsm.it (R.C.); 3 Public Health Consulting Group, Lugano 6900, Switzerland; 4 Division of Infection, Royal London Hospital, Barts Health NHS Trust, London E1 2ES, UK; simon.tiberi@bartshealth.nhs.uk; 5 Pediatric Highly Intensive Care Unit, Department of Pathophysiology and Transplantation, Università degli Studi di Milano, Fondazione IRCCS (Istituto di Ricovero e Cura a Carattere Sceintifico) Ca’ Granda Ospedale Maggiore Policlinico, Milan 20122, Italy; susanna.esposito@unimi.it; 6 Pneumology Unit, Fondazione Maugeri, IRCCS (Istituto di Ricovero e Cura a Carattere Sceintifico), Tradate 21049, Italy; antonio.spanevello@fsm.it; 7 Department of Clinical and Experimental Medicine, University of Insubria, Varese 21100, Italy

**Keywords:** MDR-TB, XDR-TB, carbapenems, imipenem, meropenem, ertapenem, effectiveness, safety, tolerability

## Abstract

Background: Carbapenems (ertapenem, imipenem, meropenem) are used to treat multidrug-resistant (MDR-) and extensively drug-resistant tuberculosis (XDR-TB), even if the published evidence is limited, particularly when it is otherwise difficult to identify the recommended four active drugs to be included in the regimen. No systematic review to date has ever evaluated the efficacy, safety, and tolerability of carbapenems. Methods: A search of peer-reviewed, scientific evidence was carried out, aimed at evaluating the efficacy/effectiveness, safety, and tolerability of carbapenem-containing regimens in individuals with pulmonary/extra-pulmonary disease which was bacteriologically confirmed as M/XDR-TB. We used PubMed to identify relevant full-text, English manuscripts up to the 20 December 2015, excluding editorials and reviews. Results: Seven out of 160 studies satisfied the inclusion criteria: two on ertapenem, one on imipenem, and four on meropenem, all published between 2005 and 2016. Of seven studies, six were retrospective, four were performed in a single center, two enrolled children, two had a control group, and six reported a proportion of XDR-TB cases higher than 20%. Treatment success was higher than 57% in five studies with culture conversion rates between 60% and 94.8%. Conclusions: The safety and tolerability is very good, with the proportion of adverse events attributable to carbapenems below 15%.

## 1. Introduction

With over 480,000 new multidrug-resistant tuberculosis (MDR-TB, *i.e.*, tuberculosis caused by *Mycobacterium tuberculosis* strains resistant, *in vitro*, to at least isoniazid and rifampicin) cases and 190,000 deaths estimated to have occurred in 2014 by the World Health Organization (WHO), tuberculosis is a global clinical and public health priority [1].

Our capacity to diagnose MDR-TB cases remains limited (one in three) and that of treating them even lower (one in four) [1].

Half of the 123,000 cases of MDR-TB reported by WHO in 2014 occurred in India, the Russian Federation, and South Africa. As of today 3.3% among the new TB cases (e.g., those never treated for TB for more than 30 days) and 20% of the previously-treated TB cases harbor MDR-TB strains of *Mycobacterium tuberculosis*. Unfortunately, about 10% of these strains meet the criteria defining extensively drug resistant TB (XDR-TB), e.g., additional resistance to at least one fluoroquinolone and a second line-injectable drug (amikacin, capreomycin, and kanamycin). In some countries belonging to of the former Soviet Union, MDR-TB hot spots exist, with reported prevalence as high as 29% in Belarus, 15% in Latvia, and 15% in Georgia [1,2].

As clinicians managing MDR- and XDR-TB patients in reference centers perfectly know, treating these cases is long, expensive, and complicated, and a wealth of experience in managing adverse events (which are frequent) is necessary [3,4,5,6,7,8].

WHO recommends the design of effective background regimens for MDR- and XDR-TB using a stepwise approach: active second-line drugs (based on the drug susceptibility test-DST) need to be introduced according to an order of priority based on their efficacy and safety. To simplify the procedure these drugs are classified into five groups based on a hierarchical order [7,9].

The core issue facing clinicians, is the difficulty in identifying at least four active drugs which are necessary to design an effective multi-drug regimen as per WHO guidelines [3,4,7,10,11].

When MDR-TB cases with resistance patterns “beyond XDR” have to be managed [3] new drugs (like delamanid [12,13,14] and bedaquiline [15,16,17,18,19,20]), and several re-purposed drugs, need to be taken into consideration [7,21].

Within the WHO Group 5, *i.e.*, drugs with unknown/limited evidence on efficacy and/or tolerability, linezolid [10,22,23,24,25,26,27,28], and carbapenems [29,30,31,32,33,34] are often used for this purpose.

Although as of today carbapenems (which include meropenem, imipenem, and ertapenem) are presently prescribed to manage M/XDR-TB cases, the evidence on their efficacy, safety, and tolerability is anecdotal [29,30,31,32,33,34,35,36,37,38,39,40,41,42,43].

The aim of this systematic review is to describe the therapeutic contribution, effectiveness, safety, and tolerability profile of the carbapenems (meropenem, imipenem, and ertapenem) added to a background regimen in the treatment of MDR- and XDR-TB cases.

## 2. Methods

A search of peer-reviewed, scientific evidence was carried out, aimed at evaluating the efficacy/effectiveness, safety, and tolerability of carbapenem-containing regimens in individuals with a pulmonary/extra-pulmonary disease, which was culture- and DST-confirmed as M/XDR-TB.

We used the database PubMed to identify any relevant full-text English manuscript without any time constraints until 20 December 2015. We decided to exclude conferences’ abstracts because, on the basis of the word count, the information provided is too limited when assessing the above described objectives. A search for ongoing or recently completed trials on clinicaltrials.gov was also performed.

Several key-words (*i.e.*, MDR-TB, XDR-TB, efficacy, effectiveness, safety, tolerability, carbapenem, meropenem, imipenem, ertapenem) were identified by the authors and combined to create *ad hoc* strings to retrieve the most relevant scientific articles. References’ lists of the selected papers were analyzed in order to identify articles missed by the adopted search algorithms.

The search exclusion criteria were the following:

(1) Case-reports describing fewer than 3 M/XDR-TB cases;
(2) Experimental studies on animals with TB;
(3) Reviews and editorials on carbapenems and M/XDR-TB; and
(4) Unclear/unconfirmed M/XDR-TB diagnosis of treated patients.

The carbapenems considered for the present review were the following: imipenem, meropenem, and ertapenem (there is no published data on the *in vivo* use of doripenem, biapenem, panipenem, and faropenem in TB patients). Observational and experimental studies were considered suitable for the estimation of the efficacy/effectiveness, safety, and tolerability of the carbapenems.

Several demographic, epidemiological, clinical variables were collected: epidemiological design of the study, age and sex of the enrolled patients, duration of the study, location/s of the study (mono/multi-center, university/reference/peripheral hospital, country/ies), number and characteristics of treated M/XDR-TB patients, number and characteristics of control cases if any, dosage and duration of the administered drugs, adverse events and their severity if any, treatment outcomes recorded according to the WHO classification [44], sputum smear, and culture conversion.

Two authors independently performed the search and evaluated the titles and abstracts of the records according to the selection criteria. Potentially interesting articles were downloaded and critically assessed; when they satisfied the inclusion criteria, the planned information was retrieved and collected using a pre-designed electronic template. The entire process was carried out following the guidelines of the 2009 Preferred Reporting Items for Systematic Reviews and Meta-Analysis [45].

## 3. Results

**Figure 1:**
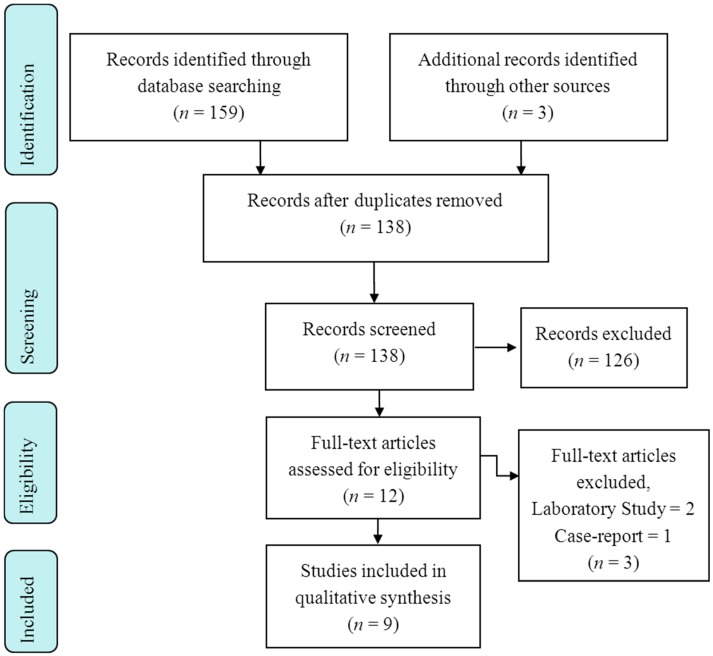
PRISMA (Preferred Reporting Items for Systematic Reviews and Meta-Analyses) 2009 Flow Diagram.

A total of 159 records were obtained from the search. Nine studies satisfied the inclusion criteria and were deemed appropriate for a qualitative analysis. The entire selection process is summarized in the PRISMA (Preferred Reporting Items for Systematic Reviews and Meta-Analyses) flowchart (Figure 1).

Two (22.2%) studies were focused on ertapenem [31,32], five (55.5%) on meropenem [29,30,40,41,43], and two (22.2%) on imipenem [33,42]. The majority of the studies were performed in Europe [29,30,31,32] (four, 66.7%), one (16.7%) in South [40] and one (16.7%) in North America [33], and three in Europe and South America [41,42,43] (Table 1).

The studies were published (or are in press) between 2005 and 2016 [29,30,31,32,33,40,41,42,43]. The epidemiological design of the studies was retrospective in eight out of nine [29,30,31,32,40,41,42,43] (88.9%) and about 50% of them (4/9) were carried out in a single medical center [30,32,33,40]. No clinical trials were performed.

Patients were recruited and exposed to carbapenems from 2001 [29] to 2015 [31,41,42,43]. The mean number of patients exposed to single carbapenems were 44.6 per study, ranging from five [31] to 96 [41,43]. Only two studies enrolled children [30,32], and three studies included a control group [29,41,42]. The proportion of XDR-TB cases was higher than 20% in all but one study [32] (Table 2).

Daily dosages of meropenem varied in the selected studies; however, all the individuals with MDR-TB exposed to ertapenem were administered a single daily dose of 1 g [31,32].

Overall, the efficacy/effectiveness profile of the carbapenems was positive. Treatment success was higher than 50% in seven studies [31,32,33,40,41,42,43], up to 80% in patients exposed to ertapenem-containing regimens [31,32] (Table 3). In particular, culture conversion rates ranged from 60.0% [31] to 94.8% [41]. Safety and tolerability was very good, with the proportion of adverse events attributable to carbapenems below 15%.

## 4. Discussion

Aim of this systematic review is to describe the effectiveness, safety, and tolerability profile of carbapenems added to a background regimen, in the treatment of MDR- and XDR-TB cases.

Overall, the results of the systematic review show that carbapenems are safe and likely to be effective in treating M/XDR-TB.

Although not all the studies reported sputum smear and culture conversions rates, the available information confirms that the treatment outcomes of the cohorts treated with carbapenems was in general better than those reported in the literature [3,4,46].

In the largest cohort of MDR-TB cases available in the literature (almost 10,000 cases) the treatment success in the whole cohort was slightly higher than 60%, being much lower in XDR-TB cases: 43% in those meeting strictly the definition (e.g., being resistant to at least one fluoroquinolone and one second-line injectable drug), 30% among those with resistance to two second-line injectables and as low as 19% among those with additional resistance to ethambutol and pyrazinamide. In the cohorts in which carbapenems were used the treatment success ranged from 57.3% to 80.3% (although the definition was not standardized for the selected studies), and the culture conversion rates (which are considered predictors of success) ranged from 60% to almost 95%.

As recently shown [46], high sputum smear and culture conversion rates are likely to indicate low probability of acquiring drug resistance.

This systematic review highlights the observational nature of the studies performed to date. In the majority of the cases the authors of the selected studies carried out a description of case-series, excluding a control group from the epidemiological design. Furthermore, sample sizes were frequently small, on the basis of being mono-center studies and being performed in areas of low M/XDR-TB prevalence. All the studies were focused on difficult-to-treat cases. Evidence on children is limited and deserves further investigation, as well as a more accurate detailed profile on the safety and tolerability profile of the prescribed drugs. In particular, it would be important to assess the causal relationship between the occurrence of an adverse event and a specific drug. The majority of the papers we retrieved do not follow an international, standardized approach for the classification of adverse events, being more focused on effectiveness.

Another important topic which should be better analyzed by future studies is the patients’ adherence to the prescribed drugs. No relevant information was provided by the studies included in this systematic review.

One of the most interesting findings is represented by the high effectiveness in cohorts of patients where the number of XDR-TB cases is high. It is clear that these preliminary results should be confirmed in order to better understand how and in which patient categories the carbapenem-containing regimens may make a difference.

Consecutive enrolment, missing blindness, and treatment randomization represent relevant methodological biases which may hamper the inference of the findings of the available studies. However, this review summarizes the current scientific evidence, highlighting the shortcomings which could be improved in the near future. In particular, more emphasis should be given to regimens including meropenem and ertapenem.

Unfortunately, it was not possible to perform a meta-analysis. Data collected and summarized from the authors of the selected papers were heterogeneous and not sufficient to carry out a quantitative analysis. Moreover, only a few papers described the clinical activity of the three single molecules, including five on meropenem, two on ertapenem, and three focused on imipenem. Likely, the authors did not compute the necessary sample size to reduce the β error and to increase the statistical power of the study. All the methodological limitations have undoubtedly reduced the reliability of the clinical findings.

Randomized, controlled clinical trials should be implemented in order to elucidate the real therapeutic contribution of carbapenems in TB treatment success; different anti-TB regimens, as well as the different duration of the carbapenem’s exposure, can create background noise which can hinder the interpretation of the carbapenem’s contribution. The experimental studies should be based on the comparisons between the different carbapenems, as well as between the carbapenem and the current therapeutic alternatives. The investigators should consider all the demographic, epidemiological, microbiologic, and clinical characteristics, including the number of resistances to anti-TB drugs, which could influence the real impact of the pharmacodynamic action of the carbapenems. Furthermore, drug-drug interactions of carbapenems with the new anti-TB drugs bedaquiline and delamanid in terms of reduced/increased efficacy and safety should be investigated.

Of interest, there are two recent early bactericidal activity trials (phase-two trials) testing carbapenems; one trial evaluates the early bactericidal activity, safety, and tolerability of meropenem plus amoxycillin/CA and faropenem plus amoxycillin/CA in adult patients with newly-diagnosed pulmonary tuberculosis (NCT02349841) has recently been completed (results awaiting).

The second trial evaluating the early bactericidal activity study of faropenem (with amoxicillin/clavulanic acid) in patients with pulmonary tuberculosis is currently recruiting (NCT02381470). These two phase-two trials will be of interest as they will be able to evaluate the individual bactericidal contribution of the penem/carbapenem drugs [47].

Economic analysis studies will also provide a proper perspective of the future role of carbapenems in the treatment armamentarium. The identification of new and more effective therapeutic strategies is crucial to reduce the increasing clinical, economic, and financial burden associated to the emergence of the MDR-TB epidemic, particularly in low- and middle-income countries (e.g., Southeast Asia and former Soviet Union countries) [48].

## 5. Conclusions

The systematic review is a contribution toward the best possible use of carbapenems when the number of active drugs necessary to design an effective regimen is lacking [3,4,9,49].

Although new drugs are becoming available to support the move towards TB elimination [50,51,52,53,54], repurposed drugs, like the carbapenems, exemplified by their tolerability, safety, and activity show a potential role in the TB treatment arena, and could be included in new TB drug combination trials, as better evidence is needed.

## Figures and Tables

**Table 1:** Epidemiological characteristics of the selected studies.

First Author	Publication Year	Country	Study Design	Clinical Setting	Study Duration
Chambers H.F. [33]	2005	USA	Prospective	Monocenter, university clinical research center in collaboration with a general hospital	ND
Palmero D. [40]	2015	Argentina	Retrospective	Monocenter, university medical centre	2012–2013
De Lorenzo S. [29]	2013	Italy, The Netherlands	Retrospective, case-control	Multicenter, university medical center and general hospital	2001–2012
van Rijn S.P. [32]	2016	The Netherlands	Retrospective	Monocenter, university medical center	2010–2013
Tiberi S. [31]	2016	Italy, The Netherlands	Retrospective	Multicenter, university medical center and general hospital	2008–2015
Tiberi S. [41]	2016	Italy, The Netherlands, Belgium, United Kingdom, Greece, Peru, Brazil, Spain, France, Ecuador, Belarus, Slovakia	Retrospective, cohort	Multicenter, university medical centers and general hospitals	2003–2015
Payen M.C. [30]	2012	Belgium	Retrospective	Monocenter, university medical centre	2009–ND
Tiberi S. [42]	2016	Italy, The Netherlands, Belgium, United Kingdom, Greece, Peru, Brazil, Spain, France, Ecuador, Belarus, Slovakia	Retrospective, cohort	Multicenter, university medical centers and general hospitals	2003–2015
Tiberi S. [43] *	2016	Italy, The Netherlands, Belgium, United Kingdom, Greece, Peru, Brazil, Spain, France, Ecuador, Belarus, Slovakia	Retrospective, cohort	Multicenter, university medical centers and general hospitals	2005–2015

ND: Not Declared: * Cohorts enrolled in the studies [41,42].

**Table 2:** Clinical features of the selected studies.

First Author	Individuals Exposed to Carbapenems	Paediatric Patients	Control Group	XDR-TB Cases (%)	Carbapenem Administered	Carbapenem Dosage
Chambers H.F. [33]	10	No	No	2/10 (20.0)	Imipenem	1 g bid
Palmero D. [40]	10	No	No	4/10 (40.0)	Meropenem	2 g tid, then 2 g tid
De Lorenzo S. [29]	37	No	Yes	9/37 (24.3)	Meropenem	1 g tid
Van Rijn S.P. [32]	18	Yes; ND	No	ND	Ertapenem	1 g qd
Tiberi S. [31]	5	No	No	2/5 (40.0)	Ertapenem	1 g qd
Tiberi S. [41]	96	No	Yes	47/96 (49.0)	Meropenem	1 g tid (2 g tid in Belgium)
Payen M.C. [30]	6	1/6	No	6/6 (100.0)	Meropenem	2 g tid, then 2 g bid
Tiberi S. [42]	84	No	Yes	57/84 (67.9)	Imipenem	500 mg qid
Tiberi S. [43] *	84 Imipenem 96 Meropenem	No	No	57/84 (67.9) Imipenem 47/96 (49.0) Meropenem	Imipenem Meropenem	Imipenem: 500 mg qid Meropenem: 1 g tid (2 g tid in Belgium)

qd: once a day; bid: twice a day; tid: thrice a day; qid: quarter: four times a day; ND: not declared; XDR_TB: extensively drug-resistant tuberculosis; * Cohorts enrolled in the studies [41,42].

**Table 3:** Effectiveness, safety, and tolerability profiles of carbapenems in the selected studies.

First Author	Sputum Smear Conversion (%)	Sputum Culture Conversion (%)	Treatment Success (%) **	Adverse events Attributed to Carbapenems (%)	Interruption of Carbapenems Due to Adverse Events (%)
Chambers H.F. [33]	ND	7/9 (77.8)	7/10 (70.0)	ND	ND
Palmero D. [40]	ND	8/10 (80.0)	3/6 (50.0)	0/10 (0.0)	ND
De Lorenzo S. [29]	28/32 (87.5)	31/37 (83.8)	ND	5/37 (13.5)	2/5 (40.0)
van Rijn S.P. [32]	ND	15/18 (83.3)	15/18 (83.3)	2/18 (11.1)	3/18 (16.7)
Tiberi S. [31]	3/5 (60.0)	3/5 (60.0)	4/5 (80.0)	0/5 (0.0)	2/5 (40.0)
Tiberi S. [41]	55/58 (94.8)	55/58 (94.8)	55/96 (57.3)	6/93 (6.5)	8/94 (8.5)
Payen M.C. [30]	5/6 (83.3)	5/6 (83.3)	ND	0/6 (0.0)	0/6 (0.0)
Tiberi S. [42]	51/64 (79.7)	46/64 (71.9)	34/57 (59.7)	3/56 (5.4)	4/55 (7.3)
Tiberi S. [43] *	Imipenem 51/64 (79.7) Meropenem 55/58 (94.8)	Imipenem 46/64 (71.9) Meropenem 55/58 (94.8)	Imipenem 34/57 (59.7) Meropenem 55/71 (77.5)	Imipenem 3/56 (5.4) Meropenem 6/93 (6.5)	Imipenem 4/55 (7.3) Meropenem 8/94 (8.5)

ND: Not Declared: * Cohorts enrolled in the studies [41,42]; ** Definition of treatment success was that provided by the authors when available.
